# Lack of access to mental health services contributing to the high suicide rates among veterans

**DOI:** 10.1186/s13033-017-0154-2

**Published:** 2017-08-18

**Authors:** Ronald D. Hester

**Affiliations:** Health Care Administration, Nicole Wertheim College of Nursing & Human Services, Florida International University, Miami, FL 33199 USA

**Keywords:** Veterans health care, Veterans suicides, Mental health care, Crisis intervention services

## Abstract

The United States has become a country that is constantly at war. This situation has created a crisis amongst our veterans. The current uneven access to appropriate mental health services that returning U.S. veterans encounter echoes the disparities in access to quality mental health services for the general population. The information presented here shows that the shortcomings of our health care system in addressing the mental health needs for our returning veterans may lead to the high suicide rates. Addressing the problem of inadequate access to quality mental health services is critical in any efforts to reforming the U.S. health care system. Our findings suggest that mental health disparities are often a leading factor to the high suicide rates among veterans who experience depression and Post-Traumatic Stress Disorder. To improve the health and well—being of our veterans who have served this nation, requires a collaboration between public and non—profit mental health providers at the State and local levels. It is imperative that we increase the availability of crisis intervention and mental health services for all veterans that have served this nation.

## Background

Many recent reports have identified that individuals enlist for many reasons, often due to patriotism, educational benefits, a family tradition of military service and financial inducements. [1].This may help explain why young adults enlist in the armed forces. Many youth often believes that they are invincible and one never thinks that they could get killed or seriously injured in a combat zone. Many recruits are high school graduates with limited job prospects and the military seems like a place to get a job and learn some skills. The reality is markedly different. Our soldiers today fight wars unlike any others who have fought. They fight a largely unseen enemy and face casualties from IEDs and suicide bombers. Many are injured and maimed by unseen foes. They fight for unclear objectives and end up coming home with limited skills and in many cases with severe physical and mental injuries. They are often separated from the military service with questionable employment prospects. A number of veterans experience depression, loss of purpose present, in some cases, an overwhelming family crisis. Their mental health difficulties profoundly touch the lives of the U.S. general public.

Recent reports documents that military personnel have experienced conditions that may have affected their mental well-being [2]. Their efforts to gain access to quality psychological health services after multiple deployments are often met significant obstacles. This lack of access to critical mental health services may led to suicidal behavior, especially among young military veterans who have completed multiple deployments to Afghanistan and Iraq. Recent data on suicide rates among Army veterans, reported by the Department of Defense (DOD), showed an increase of more than 18% from 2011 to 2014 [3]. The Department of Veteran Affairs (DVA) is now struggling to find solutions to this national crisis for our veterans.

The rate of suicidal deaths is considerably high in the veteran populations. For example, the rate of suicides among women veterans is 35 per 100,000, a rate that is much higher than their civilian counterparts [2]. Suicide in civilian populations is addressed, for example, by community-based mental health treatment providers such as Baltimore Crisis Response Inc. (BCRI), which serves the Greater Baltimore region, through a Crisis Response Hotline; mobile crisis response teams; and mental health and substance abuse treatment beds for inpatient treatment services. These and similar suicide prevention programs have proven to be successful in lowering suicide rates for the civilian population in urban communities such as Baltimore, and Boston.

These resources are not readily available at most VA Hospital and Health Systems due to the shortage of critical mental health personnel and the general lack of support in addressing crisis-intervention issues. As a result, veterans who do not rely primarily on the VA health care delivery systems to address their mental health needs once they return home from combat, do not have easy access to these critical crisis-intervention services [4].

In the civilian population, the disparities in mental health treatment often stem from the lack of mental health coverage in employment-based health insurance plans. Except for employers that sponsor health insurance plans for low-wage employees, mental health benefits are not covered under most managed care health plans provided by small businesses [5]. Many low-wage earners cannot afford a supplemental health insurance plan that would include a comprehensive mental health benefit to address crisis-intervention needs of family members who may experience depression, anxiety, and Post-Traumatic Stress Disorder (PTSD); substance abuse; and difficulties with anger management. As a result, they are not covered for crisis-intervention needs that affect many veteran families during this time of social and economic stress [6]. Consequently, when a mental health crisis occurs, these families must rely on public-supported programs funded under the State Mental Health Services Block Grant program (Table 1).

**Table 1:** Suicide rates by sex and calendar year, suicide rate (per 100,000 person—years)

Calendar year	Total	Males	Females
2014	39.0	41.6	16.7
2013	38.8	41.5	14.4
2012	38.0	40.4	16.0
2011	38.9	41.3	16.3
2010	36.3	38.5	15.4
2009	37.0	39.3	14.8
2008	38.4	40.4	14.3
2007	35.3	37.3	12.5
2006	35.9	38.5	9.0
2005	34.9	36.8	14.7
2004	35.9	38.0	13.3
2003	34.9	37.2	10.7
2002	38.0	41.7	11.7
2001	39.9	42.6	14.4

Source: U.S. DVA, Office of Suicide Prevention, 2016

The rate of suicides among users of VHA services have remained relatively stable in recent years

In Florida, a new mental health law was established in 1972, called the Baker Act. This Florida Public Law was established to enable families and loved ones to gain access to emergency mental health services and temporary detention for individuals impaired because of a mental illness. This law allows the family to assist their family members, who have experienced a mental health episode to gain the help that they need in the form of mental health treatment services. This type of program is needed in other States, to address the needs of mental health patients who are veterans, and the general public.

Many members of our military have experienced mental health problems prior to entering the military that were not treated. Once these individuals enter the military, their mental health conditions are often not detected or untreated [7]. As a result, when they re-enter society as veterans these mental health conditions may have intensified due to combat stress and PTSD. Other conditions such as combat injuries, depression, unemployment, financial stress, alcoholism, and the inevitable family discord contribute to the higher rates of mental illness.

Consequently, an increasing number of our veterans are now homeless, experiencing substance abuse problems and gambling addictions, which often lead to suicide attempts and even death [8].

The Affordable Care Act does not address the issue of expanding mental health coverage and benefits for low-wage earners under the new health plans that are available to them in recent years [9]. Not requiring *mental health* benefits as part of the mandated health benefit package is considered one of the weaknesses of the new health care law. Mental health coverage is still a great hurdle for millions of Americans at a time when various approaches to health care reforms are being considered. Many of the reforms being considered would increase out-of-pocket cost and lower benefits for many veterans. Because of the excessive cost of providing comprehensive mental health benefits under existing employer-sponsored health plans for returning veterans, these benefits are often excluded. Thus, many Americans who experience mental health problems have no access to health insurance coverage to pay for their mental health treatment [10].

The mental health crisis is a major dilemma for a growing number of Americans. The American Mental Health Association (AMHA) reported that at least 20% of all Americans are uninsured for mental health services and must rely on public hospitals to receive mental health services to address primary-care crisis intervention needs for themselves and family members. This lack of basic mental health benefits in the general public occurs at a time of mental health crisis exacerbated by the large numbers of veterans returning home from combat and often experiencing depression, substance abuse and family crisis.

Schoembaum and Kessler [11] examined common mental health disorders among Army participants and whether the disorder developed prior to entering the Army. They found in their landmark study that the most common disorders for Army participants was ADHD and intermittent explosive disorders, both are mental health predictors for suicide and accidental death based upon the results from the Army Study to Assess Risk and Resilience in Service members (Army STARRS).

The crucial issue of mental health care for veterans is more important than ever before due to the considerable number of veterans returning from combat missions who have experienced episodes of PTSD and other mental health conditions. More than 1.5 million of the 5.5 million veterans seen in VA hospitals had a mental health diagnosis in 2016. This represents about a 31% increase since 2004 [12]. Diagnosis of PTSD is on the rise, as the changing nature of warfare increases the chance for injuries that affect mental health and as our veterans face significant challenges upon returning home [13]. The potential negative effects of mental health issues, such as homelessness and suicide, affect the more than 107,000 veterans who are homeless on any given night. Current data reports that on average at least 21 veterans die by suicide each day, which makes the response to veteran mental health needs more urgent with each and every day [14].

To address this challenge, the VA has significantly invested in our mental health care workforce, hiring more than 6000 new mental health care workers since 2005. On August 31, 2012, President Obama signed an Executive Order to direct the VA to expand health manpower resources by encouraging collaboration arrangements with nonprofit organizations to work with the VA in their communities to expand the availability of health professionals by 2013, to address the problem of suicide among veterans.

President Obama signed into law The Suicide Prevention for American Veterans Act of 2015. This law requires an independent review of all Veteran Administration and Department of Defense programs aimed at preventing suicides, creates per review support and community outreach pilot programs and forms a program to repay loans debts for psychiatry students to incentivize them to work for the Veteran Administration Health System. It also creates a website to provide veterans with information about mental health services and allows the VA Health System to collaborate with non-profit mental health organizations on suicide prevention.

The challenges facing the VA are very complex and only one-third of our veterans are in the care of VA Hospitals and Health Systems [6]. Those who are employed often choose to use their private health insurance plans rather than the VA system. Veterans who are unemployed, a percentage that was recently reported at 5%, often experience the shortcoming of our health care system, which may be a contributing factor to high suicides rates.

The Veterans Administration needs to develop a new strategy with the focus on crisis intervention prevention. The existing strategy major focus is on the development of a hot-line to allow veterans to communicated with an individual, who may not be a mental health expert to assist them to consider other options than suicide. I would suggest the following crisis intervention strategy to address this problem by the Veterans Administration and the Department of Defense:

1. Establish a 30 day exist period once they are discharged to offer the each veteran, job counseling, drug prevention education, housing support and marriage counseling;
2. Establish mental health and substance abuse treatment beds for inpatient drug treatment at each regional Veteran Medical Facility; and
3. Establish mobile crisis response teams in each regional Veteran Medical Facility.
  This program has proven successful in Baltimore and other communities to address suicide prevention in the general community.

## Conclusions

It has been widely reported that the VA needs to do a better job of developing strategies for routine mental health screening and early intervention for all service members before they return to civilian life. This effort would entail identifying the several signs and symptoms that veterans may display prior to attempting suicide: (1) depression, (2) sleeping poorly, (3) losing weight, (4) telling family members they feel like a burden on their spouse, (5) drinking, and (6) using drugs. Given that this information often provides a clearer picture of potential mental disorders and indications that a veteran may be contemplating suicide, a plan of intervention based on these signs could be the first step for a crisis intervention team to provide needed assistance and conduct a psychiatric evaluation.

With a volunteer military force, a very small segment of the general population—estimated at only 2%—participates in the military. As a society, we do not experience the brunt of the hardship of losing a loved one when a veteran has committed suicide. We must do more to reach out to the veterans who are served by the VA Medical System and those that are currently not being served by the Veterans Administration System, but by our private and public health care system, to ensure that they get the help that they need.
